# Involving patients in research during a pandemic

**DOI:** 10.1111/hex.13079

**Published:** 2020-06-28

**Authors:** Carolyn A. Chew‐Graham

**Affiliations:** ^1^ School of Primary, Community and Social Care Faculty of Medicine and Health Sciences Keele University Keele Staffordshire UK

Patient and Public Involvement and Engagement (PPIE) in research is expected by funders and Research Ethics Committees.^1^, ^2^, ^3^

The National Institute for Health Research (NIHR), in the United Kingdom, states: *Every day, patients, service users, carers and the public make a difference to health and social care research and our work. So much so, that our pioneering partnership with the public has become one of the hallmarks of NIHR and is considered to be world‐leading.* ^4^ NIHR emphasizes that the input of patients, carers and the public when designing, implementing and evaluating research makes studies more effective, more relevant and often more cost‐effective.

In this issue of HEX, Berzins and colleagues report a qualitative study in which service users and carers were invited to reflect on their experiences with safety and harm in mental health services. The authors map their results onto an existing model, and the importance of communication stands out. It is of note that the interviews were conducted by the researchers using the telephone, rather than face‐to‐face, which is often held up as the optimum method to use. However, Block and Erskine^5^ suggest that interviewing by telephone has clear and distinct advantages, including providing researchers with flexibility and access that is unavailable through traditional methods. Being interviewed by telephone might also allow participants a level of disclosure that is not comfortable when an interview is conducted face‐to‐face.

Also in this issue of HEX, Raynor and colleagues describe an experience‐based co‐design (EBCD) process, within a trial, whilst Madden and colleagues describe using co‐production methods in the development of a complex intervention. Both methods are not without challenges^6^ which are likely to be magnified if the processes cannot be conducted face‐to‐face. Similarly, it would be challenging to hold a ‘community jury’ as described in Thomas et al's paper virtually. Moses and colleagues, however, describe successfully holding focus groups using videoconference to enable participants to participate remotely.

We are in currently the midst of the COVID‐10 pandemic^7^ and, due to current restrictions, our usual methods for including and involving people in the research process have become redundant. The need to adapt the methods we use for, and embrace technology to facilitate, patient and public involvement in all aspects of research is immediate. We need to do things differently.

I have drawn on the experiences of people contributing to studies that I am working on; people I have spoken to have emphasized the importance to them of continuing to contribute to research, but they also described challenges encountered with the technology required. One study PPI group member reflected on their personal preference for face‐to‐face communication in providing input, but also highlighted the risk to satisfactory input when internet connections fail:

*Although not normally a supporter of IT (always prefer the personal touch,) I don't see how it can be avoided at the moment. Flexibility being the thing, the last week my internet has been very unreliable. Unable to report the fault as my ISP had closed down their lines.*

Margaret Ogden, a patient contributor to a consensus group conducted virtually, stated:

*I have to say, I was apprehensive about using virtual platforms. But the experience has turned out much better than I thought. Meetings were arranged very quickly in this short time‐frame. A big advantage was Megan's (research team member) presence – she provided excellent support and was pivotal in preparing us for the meeting. With her help I learned how use the CHAT facility… of course, you do need practice, but it was Megan who geared me up to participate.*

Margaret's perspective emphasizes the importance of the research team member making contact with the participants prior to virtual meetings, to offer technical support and help to instil confidence in the PPI contributor, making them feel comfortable to use the various platforms available.

Stephen Dent, a lay member of a trial management group, reflected on joining the meeting virtually:

*Whilst I feel more comfortable with face to face meetings, and the Trial Management Group meeting was only my second virtual meeting, I found it quite easy both technically and as a participant. This was helped by knowing in advance the roles of all the participants, having a well‐structured agenda and, most importantly, a good chairman who allowed slight pauses in between items to allow people to have their say. Being able to join the meetings virtually allows me to keep a watchful eye on progress and to give opinions on any matters concerning the participation and welfare of the patients who are involved in the trial.*

These observations provide excellent lessons for all of us involved in virtual meetings with lay members in our research. We can ensure that we provide support to our patient, carers and public contributors, particularly in learning how to use new technology (often as we are learning ourselves), and help reduce the digital divide.^9^ We are not, however, in control of the availability of good internet access, which can result in the marginalization of people living in some rural areas.^10^

As the pandemic restrictions are lifted, we have an opportunity to continue to do things differently, maximizing the use of technology where this may be the most appropriate method of ensuring patient and public involvement in research. We would welcome submissions to HEX from authors describing novel methods of patient and public involvement in their work.
